# POLICY SERIES: MOVING FORWARD NURSING HOME COALITION CALL TO ACTION: BUILDING A SYSTEM FOR RESIDENT GOALS, PREFERENCES, PRIORITIES

**DOI:** 10.1093/geroni/igae098.1214

**Published:** 2024-12-31

**Authors:** Tara McMullen, Tetyana Shippee

**Affiliations:** Chair; Discussant

## Abstract

A 2022 National Academies of Sciences, Engineering, and Medicine (NASEM) report on nursing home quality called for nursing homes to provide care aligned with residents’ goals to ensure autonomy, quality of life, and to manage risks. While there are existing structures to support care planning clinical needs, most nursing homes lack tools and procedures to systematically identify and provide care that aligns with resident goals, priorities, and preferences (GPPs). GPPs are part of the care planning process that translates into actionable interventions supported by measurement to determine if care aligns and is concordant with GPPs, and health information technology to collect GPPs. Many nursing homes are working to improve the way they gather residents’ GPPs, and develop and implement unique care plans. These efforts represent an opportunity to improve outcomes and reduce disparities. Experts representing the Moving Forward Nursing Home Quality Coalition are developing an integrated approach to these challenges. This presentation will describe the Moving Forward Nursing Home Quality Coalition and a) the purpose and structure of the new Guide for Addressing Resident GPPs; b) an evaluation that explores whether a resident’s self-directed GPPs were included in their care; c) a description of the tools that assess GPPs and the feasibility of using those tools; and d) discussion about the development of technology advancements promoting HIT readiness and person-centered care. When completed, proposed work will provide evidence of successful and feasible quality improvement approaches to care planning resident GPPs that can inform future policy changes and guidance to surveyors.

